# Effectiveness of a psycho-educational program for improving quality of life of fibromyalgia patients

**DOI:** 10.1186/1471-2474-9-2

**Published:** 2008-01-10

**Authors:** Rita Fernández, Maria T Peñarubia, Juan V Luciano, Maria E Blanco, Mónica Jiménez, Adrián Montesano, Camino Verduras, José M Ruiz, Antoni Serrano-Blanco, FibroQoL Study Group

**Affiliations:** 1 ABS Bartomeu Fabrés Anglada, DAP Baix Llobregat Litoral, Unitat Docent Costa de Ponent, Institut Català de la Salut Gavà, Spain; 2 Red de Investigación en Actividades Preventivas y Promoción de la Salud en Atención Primaria RedIAPP, Spain; 3 Sant Joan de Déu-Serveis de Salut Mental, Fundació Sant Joan de Déu, Sant Boi de Llobregat, Spain; 4 Fundació Jordi Gol i Gorina, Barcelona, Spain; 5 ABS Viladecans-2, Institut Català de la Salut, Viladecans, Spain; 6 Servei de Reumatología, Hospital de Viladecans, Institut Català de la Salut, Viladecans, Spain

## Abstract

**Background:**

Most fibromyalgia patients are seen in primary care (PC). However, the effectiveness of the treatments prescribed by general practitioners is usually minimal. The main objective of the present research is to assess the efficacy of structured psycho-educational intervention, combined with relaxation, developed to improve the quality of life of patients suffering fibromyalgia (FM). The second objective is to assess the cost-effectiveness of this multimodal intervention.

**Method/Design:**

*Design*. Randomized controlled trial with a 12-month follow-up involving two groups, one of which is the intervention group that includes patients receiving a psychoeducational program and the other the control group consisting of patients treated for FM in the usual way.

*Setting*. Three urban PC centers in the province of Barcelona (Spain).

*Sample*. The total sample comprises 218 patients (over 18 years of age) suffering FM, selected from a database (Rheumatology service-Viladecans Hospital) of patients with this illness. Only those patients introduced in the database between the years 2005 and 2007 were included in the selection. Selected patients will be asked for written informed consent to participate in the study.

*Intervention*. Multi-component program including information about the illness, counselling about physical exercise and training in autogenic relaxation. The intervention consists of nine 2-hour sessions delivered during a two-month period. The pharmacological treatment prescribed by the physician was maintained in both groups.

*Main variables*. Sociodemographic characteristics, quality of life, use and cost of healthcare and social services.

*Measures*. Quality of life is to be measured with the FIQ and the EuroQol-5D, and the use of healthcare services with an adapted version of the Client Service Receipt Inventory (CSRI). These variables will be measured before the beginning of the program (baseline) and 1, 2, 6 and 12 months later.

**Discussion:**

This research project is an attempt to demonstrate that a psycho-educational program implemented in the context of PC can produce a significant increase in the quality of life for patients with FM, as well as a decrease in the use of healthcare and social services, compared with usual care.

**Trial registration:**

NCT00550966

## Background

Fibromyalgia (FM) is a chronic illness of unknown origin that provokes intense widespread pain and fatigue and affects people in the biological, psychological and social dimensions [1-3]. The diagnosis of FM is mainly clinical and the practitioner takes into account patient history, physical examination and laboratory evaluations. The American College of Rheumatology (ACR) established two major diagnostic criteria in 1990 [4]: first, a history of widespread pain for at least three months, and second, patient reporting of tenderness in at least 11 of 18 defined tender points when digitally palpated with about 4 Kg per unit area of force. The absence of reliable diagnostic tools has generated the need for employing subjective scales to assess the pain and fatigue of these patients [2].

The prevalence of FM in the general population is about 2–4%, and it is nine times more frequent in women than men [1,3]. In PC consultations the prevalence is about 5–7% [5]. Given its high prevalence and morbidity, patients suffering FM present an elevated frequency of use index for healthcare resources.

Although the aetiology of FM is still unknown, it is probably multifactorial [6-9], which is why multidisciplinary and biopsychosocial approaches usually obtain better outcomes than symptomatic treatment alone [10-13]. The main goal of most treatments is to reduce muscular dysfunction and functional impotence caused by pain. Among pharmacological treatments, only a minority has demonstrated significant efficacy. As far as we know, there is evidence of the efficacy of amitriptyline and cyclobenzaprine, whereas studies focused on the efficacy of tramadol and selective serotonin re-uptake inhibitors have yielded only modest evidence [14].

Non-extenuating physical exercise and some relaxation therapies can also increase pain tolerance, producing a global improvement in the quality of life of fibromyalgia patients [15,16]. Gowans et al [16] assessed physical functioning and mood state 6 and 12 months after the beginning of a supervised aerobic physical exercise program. These authors observed an improvement in participants' health when the program finished and 6 months later. Taggart et al [17] reported a satisfactory and significant increase in the quality of life of fibromyalgic patients after a 6-week relaxation program.

Another key component in current treatment of FM is the education of patients concerning their illness [18-20]. The main objective of educational programs is to increase skills in coping and healthy behaviour on the part of patients suffering FM. Scientific evidence has demonstrated that intensive education programs (between 6 and 17 sessions) improve pain symptoms, fatigue, insomnia and quality of life. These improvements continue 3 and 12 months later [14]. Furthermore, educational programs increase patient autonomy, reducing at the same time the consumption of medicines and use of healthcare services. The total cost of the continuous use of healthcare services by FM patients has not been estimated in Spain, but data from the USA reveal an annual cost of $2,274 per patient.

To date, it has not been demonstrated in Spain whether a multimodal program, combining autogenic relaxation and active healthcare education, produces a significant improvement in the quality of life of patients with FM. In addition, we do not know whether a multimodal program is more cost-effective than the treatment usually provided to these patients.

With this background, then, the main objective of the present research project will be to determine whether a multi-component psycho-educational program for treating FM leads to better quality of life (measured with the FIQ and the EuroQol-5D) at 1, 2, 6 and 12 months, than the usual care provided in Catalan Primary Health Care. The specific aims are to assess the degree of pain perceived by fibromyalgia patients attending the multimodal program, as well as the total direct costs (medication consumption and visits to public or private healthcare professionals) and indirect (work days lost), and cost-effectiveness associated with this psychoeducational intervention, compared to usual care.

## Methods/Design

### Design

Controlled trial with a random allocation of participants in two alternative branches:

1. Implementation of a multimodal psycho-educational program to improve quality of life in FM (intervention group).

2. Usual care of FM (control group).

Evaluation of treatment outcomes will be carried out at the patient level, with individual evaluation at baseline and 1, 2, 6, and 12 months after the beginning of the intervention.

### Setting and study sample

Three primary health care centres (Viladecans-2, Gavà-1 and Gavà-2) in the province of Barcelona (Catalonia, Spain) voluntarily participated for the study. The sample pool (n = 530) comprises only those patients included in a FM database of the Viladecans Hospital for the years 2005 and 2006. In the event of a lack of participants, the plan is to recruit patients with FM included in the database during the year 2007.

Patients considered for inclusion will be those aged 18–75 years-old, contactable by telephone, who meet the diagnostic criteria of FM established by the ACR. The following patients will be excluded: those with a diagnosis not based on the ACR criteria, those with cognitive impairment or suffering from physical, mental/psychiatric limitations or a severe concurrent rheumatologic illness that impedes participation in the study evaluations, those who are not expected to live at least 12 months and those without schooling.

To calculate the sample size it was taken into account that we need to obtain a difference of at least 5 points in the FIQ between those that respond and those who are resistant to the intervention. The criterion of 5 points is based on two previous studies conducted with Spanish fibromyalgia patients [21,22]. A total of 109 patients per condition is needed to conduct the study, assuming an alpha risk of 0.05 and a beta risk of < 0.20 in a bilateral contrast, a standard error of 12.1 reported in a previous study [21], and a 15% dropout rate.

### Data collection and ethical aspects

First, patients that meet the inclusion criteria will be phoned by a research assistant who is a psychologist. Second, the research assistant will make an appointment with those patients that agree to participate in the study. Third, written consent will be obtained before the beginning of the study. Participants will be provided with a general overview of the aims and activities of the study. They will be also informed that they may choose to drop out at any time with the guarantee that they will continue to receive the treatment considered most appropriate by their physician. Patients in the control group will receive the treatment considered most appropriate by their physician.

Results will be monitored and most of data collected by means of standard questionnaires conducted in face-to-face interview by the research assistant, who has been trained in the administration of the instruments. The research assistant does not know which study group the patients being interviewed belong to ("blind"). Follow-up assessments shall take place at 1, 2, 6 and 12 months after the inclusion of the patient (see Table 1).

**Table 1:** Follow-up assessments.

**Month 1**
• FIQ
• Sociodemographic variables: 1 question (current work situation)
• Pharmacological and non-pharmacological treatment received during the last month
**Month 2**
• FIQ
• Sociodemographic variables: 1 question (current work situation)
• Pharmacological and non-pharmacological treatment received during the last month
**Month 6**
• FIQ
• Sociodemographic variables: 1 question (current work situation)
• Pharmacological and non-pharmacological treatment received during the last four months
• CSRI-adapted version: use of healthcare and social services during the first six months (0–6 months)
• EuroQoL-5D
**Month 12**
• FIQ
• Sociodemographic variables: 1 question (current work situation)
• Pharmacological and non-pharmacological treatment received during the last six months
• CSRI-adapted version: use of healthcare and social services during the first six months (6–12 months)
• EuroQoL-5D

### Variables and instruments of measurement

The study protocol was approved by the research ethics committee of the Foundation Jordi Gol i Gorina (Barcelona) and consists of the following materials:

1. *Sociodemographic characteristics*: Gender, birth date, address, marital status, education level, work status.

2. *History of the illness*: age of onset, date of the first diagnosis, pharmacological treatments (oral antidepressants, benzodiazepines-hypnotics, muscular relaxants and non-steroid analgesic and anti-inflammatory medications) and non-pharmacological treatments (physical exercise, relaxation techniques, cognitive-behavioural therapy) received during the previous 6 months.

3. *Questionnaire on chronic medical condition*.

4. *Fibromyalgia Impact Questionnaire (FIQ-Spanish version) *[23]: Instrument that measures physical function, work, and well-being, and contains visual analogue scales (VAS) for pain, sleep, fatigue, stiffness, anxiety and depression. A total score may be obtained after normalization of some items (between 0–10) and adding them to the VAS. Total scores range between 0–80 (without job items), with a higher score indicating a negative impact.

5. *EuroQoL-5D questionnaire (EQ-5D – Spanish version) *[22]: Generic instrument of health-related quality of life. It has two parts: Part 1 records self-reported problems in each of five domains: mobility, self-care, usual activities, pain/discomfort and anxiety/depression. Each domain is divided into three levels of severity corresponding to no problems, some problems, and extreme problems. Part 2 records the subject's self-assessed health on a Visual Analogue Scale (VAS); it is a vertical 10 cm line on which the best and worst imaginable health states score 100 and 0, respectively.

6. *Client Service Receipt Inventory – adapted (CSRI – Spanish version) *[24]: questionnaire for collecting information about use of healthcare and social care services, other economic impacts (such as time off work due to illness) and socio-demographic information. The variant used in this study was designed to collect retrospective data on service utilisation during the first and last six months after the beginning of the treatment.

### Intervention program

Participants from the intervention group will undergo a multi-component psycho-educational program that deals with FM based on available scientific evidence. It includes measures and instruments that can be employed within the Catalan Primary Health Care, whereas the participants from the control group will receive only the treatment prescribed by their physician.

The intervention program consists of nine 2-hour sessions delivered during a two month period. Participants will be allocated in groups with a maximum of 18 subjects per group. All sessions will take place at the PC centres Gava-2 or Viladecans-2. The contents of each session are displayed in Table 2.

**Table 2:** Summary of the intervention program.

**Session 1: **General information. Expectations of the patients. History of the illness. Principal and secondary symptoms in FM. Physiological mechanisms involved in the genesis of pain.
**Session 2: **Relaxation training-I
**Session 3: **Diagnosis. Pharmacological and non-pharmacological treatments. Prognosis. Current model of health care in Catalonia. Units specialized in the treatment of FM.
**Session 4: **Relaxation training-II
**Session 5: **Strategies to increase self-esteem and regulate emotions. Pain experience and recurrent invalidation. Social support (family and friends).
**Session 6: **Relaxation training-III
**Session 7: **Holistic medicine
**Session 8: **Relaxation training-IV
**Session 9: **Benefits of physical exercise in FM. Qualitative assessment of the intervention program. Resolving doubts. Discussion concerning the strengths and weaknesses of the intervention.

In order to avoid conversations concerning the psycho-educational program among patients from different groups, we will remind the participants from the intervention of the condition that they are not allowed to talk about the program with those persons that are not attending their group. Before baseline assessment, an explanatory note addressing this important issue will be delivered to the patients participating in the program.

### Forecast execution dates

Initial recruitment of patients: September 2007

Deadline for recruitment of patients: November 2008

Deadline for period of patient monitoring: December 2009

Publication of results: 2010

### Statistical analyses

Data collected will be analysed using SAS statistical analysis software (SAS Institute Inc. 1999. v8.0, 4th ed) and employing both quantitative and qualitative techniques.

First, the intervention and control groups will be compared in order to verify that there are no significant differences between them in socio-demographic terms, clinical baseline data, etc. We shall use mean and standard deviation (SD) in continuous variables and percentages in categorical variables. For comparisons we shall use the Student-T test for continuous variables and the Chi-square test for categorical variables. Non-parametric tests may also be used.

Given that we have one between-groups factor with two levels (2 Groups: Intervention and Control) and one within-subjects factor with five levels (Baseline and 4 follow-up assessments: 0, 1, 2, 6, and 12), a 2 × 5 mixed factorial ANOVA will be performed to study the effect of the psycho-educational program on the quality of life. Then, between-subjects planned comparisons may also be used (post-hoc comparisons with independent samples T-test). If we observe differences between groups in quality of life prior to treatment, one-way analyses of covariance will be carried out using baseline scores as a covariate). In addition, we shall carry out a logistic regression analysis taking the groups as dependent variable (the target variable is a categorical variable with two categories: Intervention and Control) and the collected measures as independent variables. This analysis will let us to know the association between the intervention and the cost of the healthcare resources, controlling for the rest of variables.

### Cost-utility analyses

Societal cost perspective will be used for the calculation of costs. *Direct costs *are going to be calculated by adding the costs derived from any medication and use of health-related services (general practitioner sessions, specialized medical sessions, emergency room sessions, and hospital in-patient stay). The cost of medications will be calculated by determining the price per milligram during the study, according to the International Vademecum (Red Book) 2007–2009, including value-added tax. Total costs of pharmacological treatment will be calculated by multiplying the price per milligram by the daily dose in milligrams and the number of days receiving such treatment. Costs derived from the use of health-related services will be calculated considering the SOIKOS unit costs database (25). *Indirect costs *will be calculated considering the days under sick leave and multiplying them by the minimum daily wage in Spain for 2007–2009. Finally, *total costs *are going to be calculated by adding direct and indirect costs.

In Spain, the National Healthcare Service (NHS) is financed by the general taxes levied by the state. In the municipalities where the study is carried out, it is administered by the autonomous government of Catalonia. Medical visits and hospital admissions are fully covered by the NHS. Medications prescribed are fully covered for retired persons, and partially for those still employed. Sick leave requires a physician's authorization, and patients unable to work continue receiving most of their salary.

When performing cost-utility analyses, two or more therapeutical options are compared in order to determine which one is the best for maximizing the benefits in light of the available resources [25,26]. This is achieved by calculating the relationship between the costs of a given intervention (e.g. A) and its consequences, expressed in QALYs, compared with another (e.g. B). This relative value is called incremental cost-utility ratio (ICUR), and it expresses the relationship between the costs and effects of one intervention compared with another.

**Figure 1:**
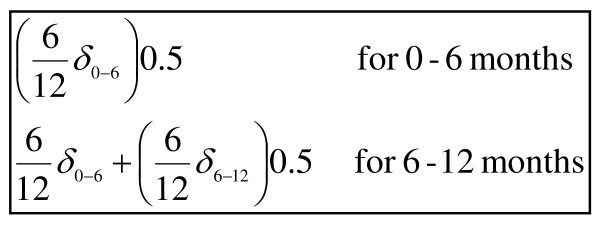
Formulas for calculating the QALYs gained in the first and last six months after the beginning of the program.

As the duration of the study is only 12 months, neither costs nor outcomes are subject to discounting [26]. Treatment costs during 12 months follow-up will be modelled by a multivariate gamma regression with a log link. Gamma modelling has been suggested as a suitable choice for analysing cost data, taking into account the skewing of the distribution of the cost data [27,28]. QALYs gained in the first and last six months after the beginning of the program will be approximated by measuring the area under the curve (Figure 1).

In the Figure 1, d_0–6 _is the incremental treatment effect on change in EQ-5D utility score for the first 6 months and d_6–12_the incremental treatment effect on change in EQ-5D utility score for the last 6 months. These incremental treatment effects will be estimated using multivariate ordinary least squares regressions, adjusting for baseline differences among treatment groups. The covariates included in the models will be: age, gender, years of education, employment and marital status, baseline FIQ and EQ-5D utility scores, and type of medication prescribed. To address uncertainty in the ICUR sampling distribution, non-parametric bootstrapping with five thousand replications will be carried out for each treatment comparison [29].

## Discussion

FM is a chronic illness that seriously alters the well-being of patients [1-3,30]. One of the main expected contributions of the present project is that we will have the opportunity to assess not only whether a multimodal psycho-educational program is feasible in primary health care, but also whether it is more cost-effective than the usual care provided for FM in this context.

Although FM has an irregular course that causes oscillations in the patients' quality of life, the random assignment of the participants to the conditions will minimize the differences not produced by the intervention. Baseline comparisons between groups will permit detection of possible pre-treatment differences.

In our opinion, the application of the program in PC entails a low outlay because nursing personnel may be trained to develop the program, as in other recent educational programs developed for a number of chronic illnesses [31,32].

## Competing interests

The author(s) declare that they have no competing interests.

## Authors' contributions

RF is the principal investigator and developed the original idea for the research. The study design was made by RF, MTP, ASB, EB, MJ, CV and JMR. These authors, with the help of JVL and AM, designed and planned the intervention that is going to be evaluated. ASB and JVL developed the statistical methods. All authors have corrected draft versions and approved the final version of the manuscript.

## Pre-publication history

The pre-publication history for this paper can be accessed here:


